# ABC Transporters 45 Years On

**DOI:** 10.3390/ijms242316789

**Published:** 2023-11-27

**Authors:** Anthony M. George

**Affiliations:** School of Life Sciences, University of Technology, Sydney, NSW 2007, Australia; tony.george@uts.edu.au

ABC transporters constitute one of the largest gene families among all species [1,2,3]. Although many of these transporters have been discovered in other species (for instance, there are over 100 in *Escherichia coli*), only 48 have been identified in humans. The first such transporter, P-glycoprotein (ABCB1), was discovered in the mid-1970s [4], and hundreds of others were subsequently identified, with functions ranging from drug resistance to antigen processing and the import and export of nutrients, toxins, lipids, and polysaccharides [5]. These primary active transporters are integrated membrane proteins, except for a small subclass involved in chromosome maintenance and protein synthesis.

All membrane ABCs share a common architecture of two transmembrane domains and two ABC cassettes or nucleotide-binding domains (NBDs), which are intracellular and power the translocation of allocrites via coordinated allosteric ATP hydrolysis in each NBD [6,7,8]. In eukaryotes, the transporters mostly are single polypeptides, while in prokaryotes, they constitute two half-transporter domains or four separate subunits. The four-domain architecture is similar, with extensive homology in the NBD cassettes, reflecting their role in binding ATP. The transmembrane domains contain the most diverse homology, and this is in keeping with the role of the transmembrane domains in translocating allocrites into or out of cells [9]. In bacteria, two additional periplasmic domains bind incoming substrates and release them to the translocating membrane domains [10]. Many excellent reviews have been written on ABC transporters [1,7,10,11,12,13,14,15].

This brief Editorial is intended to introduce the articles in this Special Issue. As with most papers on ABC transporters, the topics often vary widely, and there is a rich lode of research still to be performed. In general, human ABCs attract the most attention, as their dysfunction, over-expression, or failure to be expressed lead to a range of human medical diseases and illnesses. Before providing short introductions to the papers in this issue, the following salient questions are posed: How do ABC transporters work? What are the mechanistic steps that enable the import or export of substrates? ABCs were discovered nearly 45 years ago, with extensive research conducted on them since, thousands of papers published, and dozens of complete X-ray or Cryo-EM structures solved; however, we still cannot resolve these mechanistic questions, probably because it is virtually impossible to “freeze” the transporter in each phase or step in the transposition cycle [16]. What we can say with certainty is that there are two models of ATP hydrolysis cycling: the Switch mechanism [17] and the Constant Contact Reciprocating mechanism [18]. Though researchers are gradually leaning towards the CC Model and showing less support for the Switch Model, like the overarching mechanism, much is still to be resolved. The first-discovered ABC transporter, P-glycoprotein (ABCB1 as it is known today) is still the most studied due to its role in cancer chemotherapy failure in humans [19]. Suffice to say that five of the eight papers in this issue are concerned with ABCB1.

In this Special Issue of *Int. J. Med. Sci.*, *ABC Transporters 45 Years On*, we have assembled eight reviews and research papers that describe recent research achievements in addition to addressing questions about how ABC transporters work.

Singh et al. (contribution 1) describe a study of the synergistic inhibitory effects of quercetin and cyanidin-3O-sophoroside on ABCB1. The authors emphasise the importance of ligand–ABCB1 interactions. An earlier study by the same authors found that quercetin hampers ABCB1 activity, whereas cyanidin-3O-sophoroside stimulates ABCB1 ATPase activity and only weakly inhibits substrate transport. Together, the two molecules act synergistically to inhibit both ATPase and transport activity, presumably by shifting ABCB1 to an inward-facing conformer. In silico docking and MD simulations revealed that both compounds bound to the transporter simultaneously but at different sites. This is a paper worth reading in full.

ABCA4 is expressed in the discs of photoreceptors’ outer segments. Mutations in the ABCA4 gene are the main cause of retinal degenerations known as “ABCA4-retinopathies”. Recent research has revealed that ABCA4 is expressed in other cells, as well, such as hair follicles and keratinocytes. Ścieżyńska et al. (contribution 2) conducted the first investigation of the role of the ABCA4 gene in human keratinocytes and hair follicle stem cells, and showed that that ABCA4 gene silencing enhances the toxic effect of the all-trans-retinal effect on human hair follicle stem cells.

In another interesting study, da Costa et al. (contribution 3) focus on ABCB1 research, but link it to another early-described multidrug ABC, namely, ABCC1 (MRP-1). In addition to their originally identified multidrug resistance phenotypes, these two transporters have other non-transport roles, participating in in endo- and xenobiotic excretion, cellular detoxification, and the stress response. It is these latter roles that are investigated in this study. Metastasis originates from the epithelial–mesenchymal transition (EMT), in which cells acquire a migratory phenotype, invading new tissues. Cells undergoing EMT exhibit enhanced ABCB1 expression. ABCB1 inhibition by verapamil increased snail and fibronectin expression and the upregulation of ABCB1, evidencing coincident cell signalling pathways leading to ABCB1- and EMT-related marker transcription, rather than a direct effect of transport. For the first time, this group show that increased ABCC1 expression and activity was observed after EMT. Considering ABCC1 is involved in the stress response, affecting intracellular GSH content and drug detoxification, the authors suggest that this transporter could be used as a therapeutic target in cancer cells undergoing EMT.

Yagishita et al. (contribution 4) studied the effects of *NR112* and *ABCB1* genetic polymorphisms on everolimus pharmacokinetics in renal transplant patients. Everolimus is a mammalian target of rapamycin, which is used for the prophylaxis of acute rejection in renal transplant patients. It is also a substrate of ABCB1 in the intestines. NR112 is part of the nuclear receptor superfamily, some of which are transcription factors characterized by a ligand-binding domain and a DNA-binding domain. The NR112 protein is a transcriptional regulator of the cytochrome P450 gene. The authors suggest that their data indicate that the management of everolimus blood concentrations after administration may be more important than an analysis of drug metabolism and transport-related SNPs before everolimus administration.

Lutz Schmitt’s group (contribution 5) produced a significant review paper that combines our knowledge of the variety of ABC transporter functions with a new role in bacterial nanomachineries This review focuses on four such nanomachineries: the Mac system, which provides drug resistance; the Lpt system, which catalyses vectorial LPS transport; the Mla system, which is responsible for phospholipid transport; and the Lol system, which is required for lipoprotein transport to the outer membrane of Gram-negative bacteria.

The review by Jones and George (contribution 6) updates our knowledge of ABC structure and function, elaborates on the two models of the transduction cycle, and focuses on the contribution of structure alone to solving the mechanistic riddle of how these integral membrane proteins function as importers or exporters. This review brings ABC transporter structure and mechanistic function up to date by surveying the recent literature in order answer key questions about these transporters.

Mora Lagares and Novic (contribution 7) simplified the basic challenge of MDR. Throughout decades of study, researchers have struggled to find new therapeutic molecules to reverse MDR associated with the overexpression of ABCB1, the most extensively researched drug efflux pump. In silico models and molecular modelling may predict the interaction of compounds with ABCB1, which is of value in the early stages of drug development. This topic is reviewed by these authors in an updated review of studies and data published in the last five years.

Chai, Callaghan, and Gelissen (contribution 8) provide a comprehensive and current update on the role of P-glycoprotein in the brain. Among the many authors researching this topic, a special mention should be made of Anna Hartz (University of Kentucky), whose excellent work is cited liberally in this review. Since this is a review, it is difficult to summarise or pick out the many highlights; thus, it is suggested that it be read it in its entirety. Suffice to say that, in addition to ABCB1′s role in maintaining the integrity of the blood–brain barrier, its involvement in neurological, neuroinflammatory, and neurodegenerative conditions, including Alzheimer’s disease and epilepsy, is growing in recognition, at an apt point in time for such a review.

This Special Issue, which includes a selection of reviews and research papers on ABC transporters, shows us what most other publications in this field have discovered in the last 45 years—that these crucial proteins, in all species, are reluctant to reveal their complete transduction pathways and cycles. In particular, progress is being made in the study of MDR in cancer research and a host of physiological and pharmacokinetic functions impacted by this important family of primary active transporters.
